# Supplementary figures and images for: DA-Raf synergistically binds to the plasma membrane and Ras to suppress ERK signaling
Source: Life Sci Alliance. 2025 Oct 21;8(12):e202503300. doi: 10.26508/lsa.202503300 (PMC12540644; doi:10.26508/lsa.202503300)

Source Data for Figure 1.1

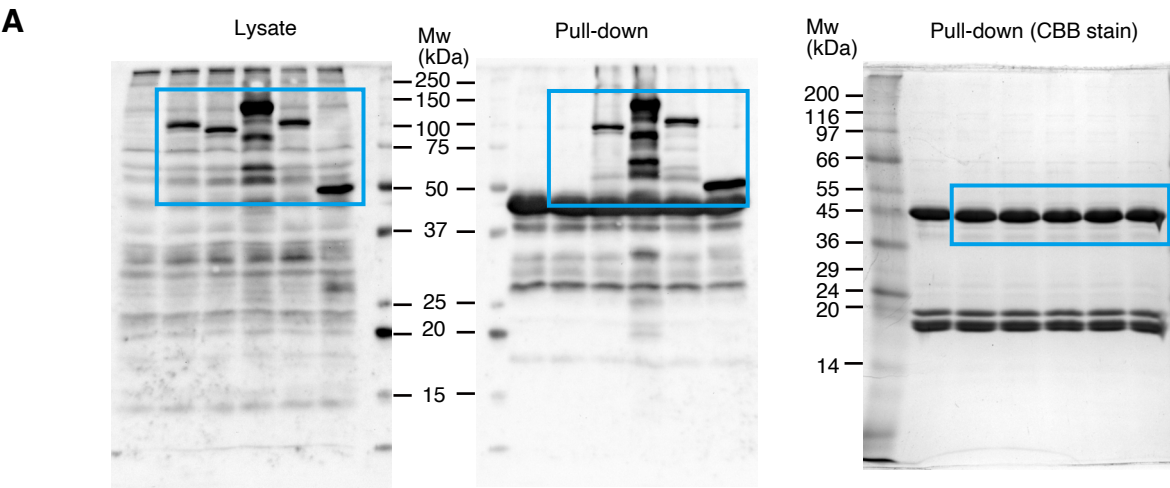

Supplement: Supplementary file 1 [file LSA-2025-03300_SdataF1.1.pdf]

Source Data for Figure 2.1

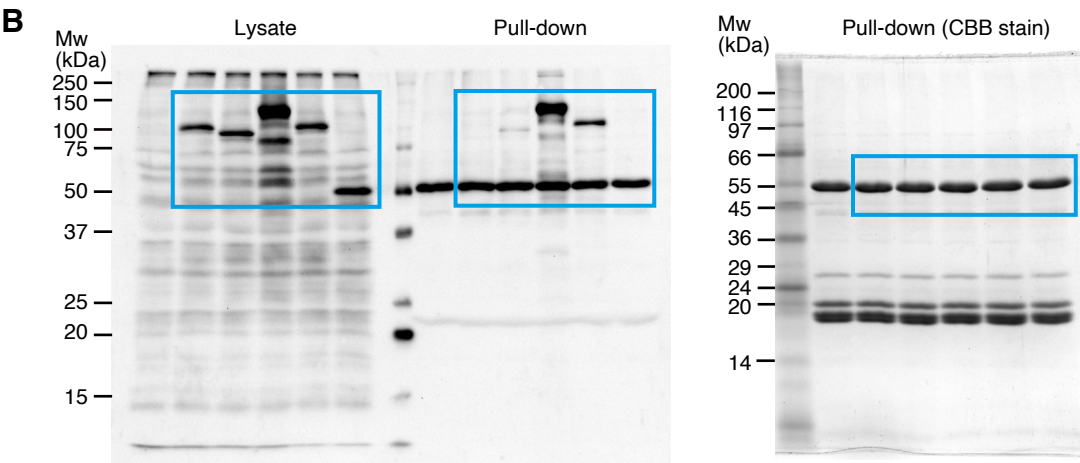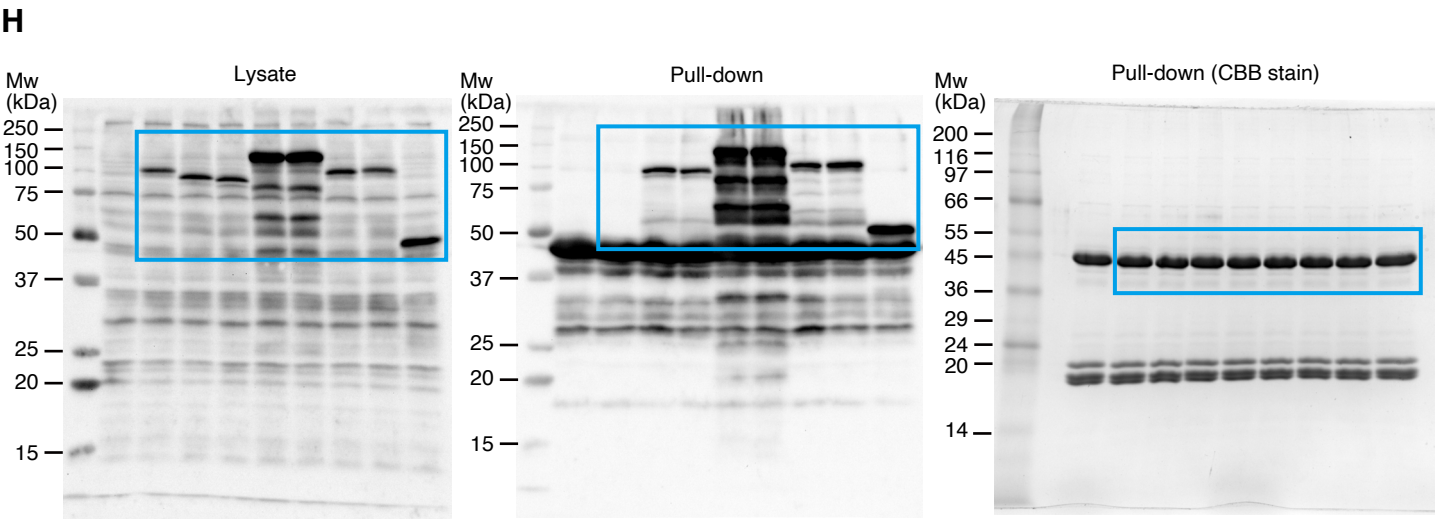

Supplement: Supplementary file 4 [file LSA-2025-03300_SdataF2.1.pdf]

Source Data for Figure 4.1

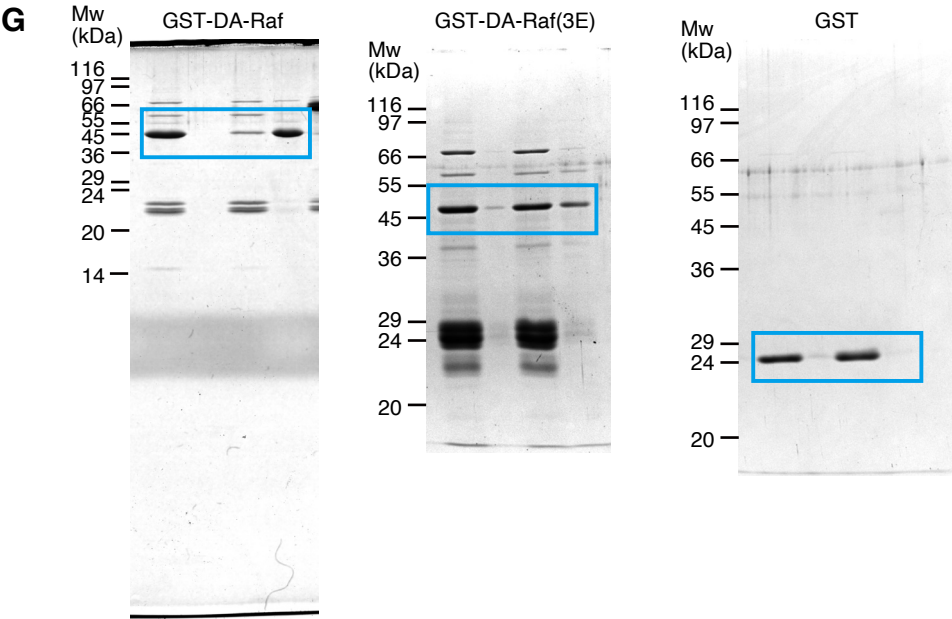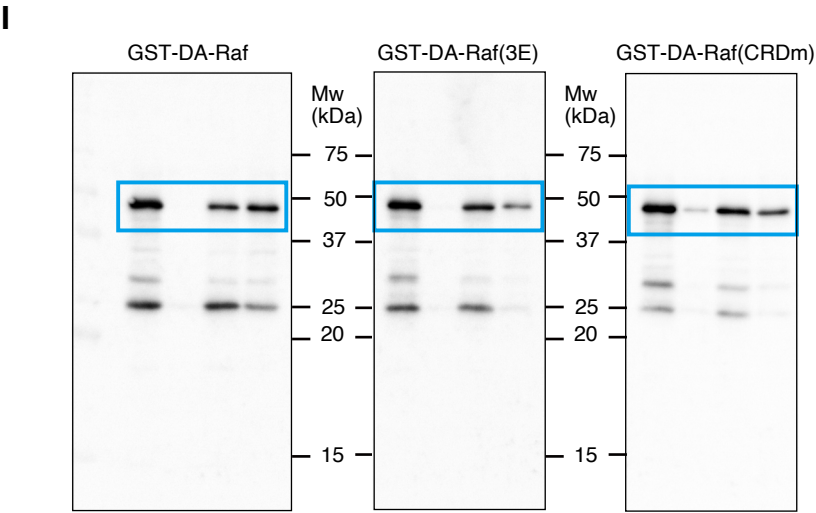

Supplement: Supplementary file 7 [file LSA-2025-03300_SdataF4.1.pdf]

Source Data for Figure 5.1

A

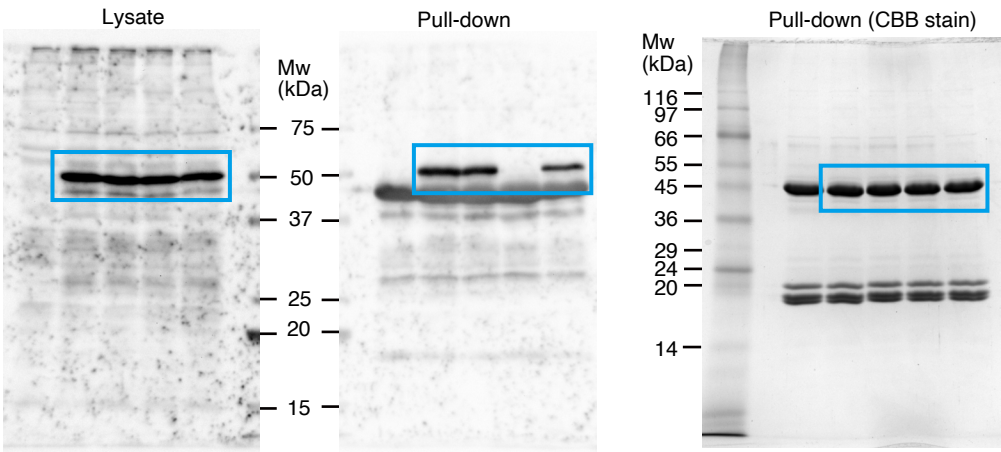

Supplement: Supplementary file 12 [file LSA-2025-03300_SdataF5.1.pdf]
